# Understanding the post-2010 increase in food bank use in England: new quasi-experimental analysis of the role of welfare policy

**DOI:** 10.1186/s12889-022-13738-0

**Published:** 2022-07-16

**Authors:** Filip Sosenko, Glen Bramley, Arnab Bhattacharjee

**Affiliations:** 1 The University of Glasgow, Glasgow, UK; 2 Heriot-Watt University, Edinburgh, UK

**Keywords:** Food insecurity, Food banks, Welfare system, Benefits

## Abstract

**Background:**

The number of food banks (charitable outlets of emergency food parcels) and the volume of food distributed by them increased multi-fold in the United Kingdom (UK) since 2010. The overwhelming majority of users of food banks are severely food insecure. Since food insecurity implies a nutritionally inadequate diet, and poor dietary intake has been linked to a number of diseases and chronic conditions, the rise in the number of people using food banks is a phenomenon of significant importance for public health. However, there is a shortage of robust, causal statistical analyses of drivers of food bank use, hindering social and political action on alleviating severe food insecurity.

**Methods:**

A panel dataset of 325 local authorities in England was constructed, spanning 9 years (2011/12–2019/20). The dataset included information about the volume of parcels and the number of food banks in the Trussell Trust network, as well as economy-related, welfare system-related and housing-related variables. A quasi-experimental approach was employed in the form of a ‘first differencing’ ecological model, predicting the number of food parcels distributed by food banks in the Trussell Trust network. This neutralised bias from omitting time-constant unobserved confounders.

**Results:**

Seven predictors in the model were statistically significant, including four related to the welfare system: the value of the main out-of-work benefit; the roll-out of Universal Credit; benefit sanctions; and the ‘bedroom tax’ in social housing. Of the remaining three significant predictors, one regarded the ‘supply’ side (the number of food banks in the area) and two regarded the ‘demand’ side (the proportion of working age population on out-of-work benefits; the proportion of working age population who were unemployed).

**Conclusion:**

The structure of the welfare system has been partly responsible for driving food bank use in the UK since 2011. Severe food insecurity could be alleviated by reforming aspects of the benefit system that have been evidenced to be implicated in the rise in food bank use. More broadly, the findings provide support for ‘Health and Health Equity in All Policies’ approach to policymaking.

**Supplementary Information:**

The online version contains supplementary material available at 10.1186/s12889-022-13738-0.

## Background

Until around 2009–10 food banks (charitable organisations distributing free food parcels) used to be a very marginal phenomenon in the UK. However, the number of food banks increased very rapidly in the 2010s. The Trussell Trust, which support the largest network food banks in the UK, had around 35 food bank centres in 2010/11, 650 in 2013/14 and nearly 1300 in 2019/20 [1, 2]. (By food bank centres we mean individual venues. A food bank may have more than one such venue). The dynamics of the volume of food aid broadly followed this trend, with 61,000 food parcels distributed in 2010/11, 0.9 m in 2013/14 and 1.9 m in 2019/20 [1, 2]. Less is known about food banks operating independently of the Trussell Trust network (estimated to represent around 40–45% of all UK food banks, many of which are members of the Independent Food Aid Network) but one study suggests that they underwent a similar growth [3].

Reliance on food banks is a symptom of household food insecurity, particularly its severe type: around 80% of people referred to food banks in the Trussell Trust network are severely food insecure [1]. ‘Household food insecurity’ is an established, internationally used concept and is defined here as ‘a household-level economic and social condition of limited or uncertain access to adequate food’ [4]. In the UK, researchers commonly classify households into four categories: food secure, marginally food insecure, moderately food insecure and severely food insecure. Broadly speaking, ‘marginal’ food insecurity signifies uncertain access to adequate food (anxiety about adequate food supplies in the near future, for financial reasons), ‘moderate’ food insecurity indicates inability to afford a balanced diet, and ‘severe’ food insecurity indicates inability to afford enough food. (In this paper, as often in relevant literature, the term ‘food insecurity’ collectively signifies the moderate and severe levels). Food insecurity is typically measured via household surveys, with respondents asked standardized questions.

The most recent survey data prior to the Covid-19 pandemic shows that 4% of UK households were severely food insecure in 2019/20, while a further 4% were moderately food insecure [5]. While these figures regard respondents’ experiences in ‘the last 30 days’, research in the United States (US) suggests that the reported levels of food insecurity would have been much higher if the questions referred to ‘last 12 months’ [6]. As it is estimated that around 2.5% of all UK households used a food bank at any point in 2019/20 [2], it follows that a substantial proportion of severely food insecure people in the UK do not use food banks (consistent with Canadian evidence [7]). Barriers to accessing food aid may take the form of a geographical distance to a food bank, its opening hours, the necessity to obtain a referral or, in the case of The Trussell Trust, the fact that it is a Christian organisation operating largely from Christian churches, which may discourage non-Christians [8]. Furthermore, some people prefer to go without food than to experience the feelings of shame and stigma associated with relying on charitable food aid [9, 10]. However, even those who use food banks should not be perceived as automatically sheltered from negative effects of food insecurity, mainly because food aid does not address the underlying causes of household food insecurity [11], meaning that it amounts to a short-term ‘sticking plaster’. Additionally, access to food aid may not be available ‘as and when’ needed, resulting in periods of lacking food. A food parcel would also often lack fresh vegetables and fruit [12] while providing disproportionately high sugar and carbohydrate [13, 14], meaning that a balanced diet still cannot be achieved.

Since food insecurity implies a nutritionally inadequate diet, it is a phenomenon of significant importance for public health. Poor dietary intake has been linked to a number of diseases and chronic conditions, including cardiovascular disease, Type 2 diabetes, some types of cancer, and osteoporosis [15, 16]. In addition, inadequate dietary intake during pregnancy and early childhood can increase the risk for birth defects, anaemia, low birth weight, preterm birth, and developmental risk [15–18].

There is also some evidence that diets with a high glycaemic index and load (e.g., diets containing high amounts of refined carbohydrates and sugars) may have a detrimental effect on psychological wellbeing [19]. Poor dietary intake also contributes to obesity, which is associated with many serious physiological, psychological, and social consequences for children and adults, including high blood pressure [20, 21], heart disease [22], diabetes [20, 21], pregnancy-related complications [23], decreased life expectancy [24], asthma [25, 26], depression [27, 28], and stigmatization [29, 30].

Since absence of food insecurity is a desired outcome from a public health perspective, the major increase in food bank use is of concern; some researchers have warned of a ‘public health emergency’ (e.g., [31]). It would be in the public interest if this situation attracted governmental and wider societal efforts at reversing the trend, by addressing the root causes of food insecurity (as opposed to handing out more food, which does not resolve food insecurity [11]). A prerequisite for an effective response is to understand the issue, and in particular to understand what the drivers are, the focus of this paper.

While demographic characteristics of people experiencing food insecurity (such as younger age, non-white ethnicity, low education, having a disability, being unemployed) have been studied [32], the body of research into drivers of food insecurity in the UK is very limited [2]. In contrast, several studies of drivers of food bank use have been conducted. They have tended to point at issues with the welfare system, particularly those resulting from changes introduced after 2010, such as tightened benefit eligibility, increased benefit conditionality and reduced generosity of benefit levels (e.g. [33]). Generally labelled ‘Welfare Reform’ (WR) and primarily justified by the need to reduce state deficit [34], these changes were targeted at working age population, which probably explains why people of pension age have been so under-represented among food bank users [1]. Key elements of WR include:

- Benefit sanctions, i.e. a temporary suspension of benefit payments due to non-compliance with requirements;
- Freezing of most benefit levels between April 2016 and March 2020 at 2015/16 rates;
- Reductions to, and limits on, Local Housing Allowance (LHA) for private tenants;
- ‘Bedroom Tax’ for under-occupation of social housing;
- Benefit Cap (limit on total amount per family);
- 5-week wait for the first Universal Credit (UC) payment, along with other features including on-line application process and the recovery of debts and advances from benefit payments;
- Replacement of Disability Living Allowance with Personal Independence Payment (PIP), with more frequent reassessments and a different eligibility structure.

Factors not directly related to the benefit system, such as job loss, ill health, homelessness, domestic violence or bereavement have also been identified as drivers of food bank use, albeit (in the assessment of some researchers) of overall smaller weight than benefit issues [1, 33, 35].

The common weakness of these studies is that the evidence they provided is long way from the ‘gold standard’ of causal research, a randomized experiment. The evidence has been either qualitative in nature – and as such dismissed by policy makers as ‘not robust’ [36] – or quantitative, but limited to description of the population of food bank users, without contrasting them with non-users of food banks (and dismissed in turn as ‘self-selecting data’ that ‘can’t prove anything’ [37]). Attempts at quasi-experimental analyses have been few and usually limited to the weakest quasi-experimental design, simple balancing on covariates by means of multiple regression conducted on cross-sectional data (e.g. [38, 39]). While these analyses found statistically significant associations between certain elements of WR and food bank use, the findings cannot be described as robust due to the possibility of the effect being biased by omitted variables. Only two studies attempted to use a stronger quasi-experimental design, where ‘fixed effects’ regression modelling has been employed to evidence the causal link between benefit sanctioning and food bank use [40, 41]. Thanks to this design these two studies neutralised bias from time-invariant omitted variables (see below). However, benefit sanctions are just one factor on a long list of potential drivers of food bank use; there is a need to investigate those other factors using as strong quasi-experimental design as possible.

## Methods

### The study

The analysis reported in this paper builds upon the analysis that formed part of Wave 1 of the *State of Hunger* study [1]. This mixed-methods research project was commissioned by the Trussell Trust and aimed to collect evidence that would help answer the question ‘what drives hunger in the UK?’. Hunger was conceptualised in terms of ‘household food insecurity’. Research methods included in-depth interviews with key stakeholders and experts, a major survey of people referred to Food banks in the Trussell Trust network, in-depth interviews with 25 respondents to that survey, a survey of food bank managers, a survey of agencies referring people to Food banks in the Trussell Trust network, and statistical modelling of drivers of changing take-up of food parcels over time. This modelling has been subsequently updated and enhanced and is the subject of this paper. While the results themselves have been published in a research report [2], the current account includes substantial unpublished parts of the analysis, as well as a discussion of the analysis strengths and limitations, and the public health implications of the results.

### Data

A bespoke panel dataset of 325 local authorities in England was constructed, tracked over nine financial years 2011/12–2019/20. The key variable that served as the dependent variable in the modelling analysis was the number of food parcels distributed by food banks in the Trussell Trust network in each local authority in each financial year, scaled by the size of the working age population. (We decided to use working age population rather than overall population because pensioners constitute a very small minority of food bank users, around 2% [1]). A large number of variables (that have been identified by previous studies or the *State of Hunger* study as potential determinants of take-up of food parcels) have been added to the dataset, from sources including the Office for National Statistics, the Department for Work and Pensions, the Valuation Office Agency and the Ministry for Housing, Communities and Local Government. These variables covered aspects of the benefit system (such as benefit levels and benefit sanctions), the structure of the local economy (such as the proportion of working age people who claim out-of-work benefits), demographic composition of the local population (e.g. proportion with a disability), local housing and homelessness situation (such as average private rent, proportion of households accepted by the local council as homeless) (see Additional file 1 for a complete list of these variables). Monetary variables were adjusted for inflation using 2011 as the basis.

### Analysis

The most undisputed approach to find proximate drivers for any phenomenon is a controlled experiment, where outcomes for some randomly chosen individuals subjected to a treatment (the treatment group) are compared to others who were not assigned to the treatment (the control group) [42]. However, a control experiment into drivers of food bank use is not possible for ethical and practical reasons. With observational data, there are three broad strategies for balancing the treatment and control groups on confounders, post-intervention [43–45]:

1. Balancing on observed confounders only. Main techniques here include multiple regression and matching. The disadvantage of these approaches is that they are vulnerable to omitted variable bias.
2. Balancing on observed confounders, on unobserved confounders that vary across units but not across time, and on unobserved confounders that vary across time but not across units. Techniques in this group are collectively named ‘fixed effects’ models.
3. Balancing on observed and unobserved confounders. The two main techniques here are instrumental variables and regression discontinuity.

Due to a lack of suitable instrumental variables and the unsuitability of the regression discontinuity design (RDD), it was decided to employ techniques from group 2 above. (RDD requires there to be a well-defined ‘intervention’ with a cut-off eligibility point on a continuous scale. None of the factors under our study met this condition). Techniques from group 2 were preferred over techniques from group 1 mainly due to the former’s better protection from omitted variable bias.

Techniques in group 2 use each unit in the dataset (in our case, each local authority) as its own control group over time, and information about variation between units (here, differences between local authorities) is discarded. The two most commonly used estimators are ‘first differencing’ (FD) estimator and ‘fixed effects’ (FE) estimator. If FE assumptions hold, the FE estimator is more efficient than the FD estimator [46]. However, a decision has been taken to choose FD as the primary modelling technique because it does not require a relatively stronger strict exogeneity assumption [46].

The analysis draws on ecological regression, which is a method of running regression on aggregates (such as averages within geographical units – here, local authorities) and interpreting the results as relations on the level of individual units (here, persons/households) [47].

### Modelling procedures

We started by compiling a ‘longlist’ of over 30 candidate explanatory variables. The selection included factors suggested by previous studies to be potential determinants of take-up of food parcels or destitution in general [10, 48, 49]. These factors covered demographic characteristics of local authorities, economy-related characteristics, housing- and homelessness-related characteristics, and welfare characteristics. Table 1 presents some of the longlisted variables and reasons for their inclusion while Additional file 1 contains the full list:

**Table 1:** Example longlisted factors potentially responsible for driving food bank parcel uptake

Variable	Reason for inclusion
Number of operational Trussell Trust food bank centres	The more venues where food parcels can be obtained (the ‘supply’), the more the ‘demand’ will be met and will translate into a higher number of food parcels collected.
Number of lone parent households	Control variable. Lone parent households have been identified by previous studies as over-represented among food bank users [1, 50].
Number of people who are non-UK born	Some people who are non-UK born, including refused asylum seekers, will lack access to the welfare safety net, due to their immigration status. Lack of access to the benefit system has been identified as a reason why some people use food banks or fall into destitution [1, 10, 50].
Real gross weekly median pay	Low pay has been identified as a reason for using food banks [1]. This variable is a proxy for the extent of in-work poverty in the local authority: a local authority with a lower-than-average median pay will have more households who are in in-work poverty than a local authority with above-average median pay.
Percent of employees working on a part-time basis	Part-time workers typically have lower income than full-time workers. Similarly to the ‘median pay’ variable above, this variable captures information about low-paid households: a local authority with more part-time workers will have more in-work poverty than a local authority with fewer part-time workers, ceteris paribus.
Number of work seekers	Being a work seeker indicates having low/no income, which in turn is the main reason for needing to use a food bank or falling into destitution [1, 33, 50].
Real value of main out-of-work benefits	The less generous the benefits, the higher the likelihood that households relying on them will need to resort to using a food bank or fall into destitution [10, 48].
Number of LHA claimants	Post-2010 changes to the LHA regime resulted in less financial support for private renters, squeezing household budgets and leading some to debt and destitution [10].
Council Tax collected by LA as proportion of all collectible Council Tax	The localisation of Council Tax Support from 2013 has led to some previously exempt households having to pay Council Tax, squeezing household budgets and leading some households to destitution or needing to use food banks [1, 10].
Number of cases of unsuccessful Disability Living Allowance to Personal Independence Payment reassessment	The loss of Disability Living Allowance indicates a drop in household income and has been identified as a reason why some households need to use food banks or become destitute [1, 10].

We subsequently examined longlisted variables to identify ones that had simultaneously a meaningful effect on the R squared, were statistically significant and had a sizeable effect on the outcome variable, or whose exclusion had a crucial impact on the coefficients of the other variables. We then fitted an initial FD model using ‘shortlisted’ variables (see Additional file 1). At this point multicollinearity was examined; some variables were collinear to a degree but not to the point where it would create issues. Next, variables that were not statistically significant at the conventional 5% level were dropped, with the exception of two variables that were deemed to be key control variables (the proportion of people on out-of-work benefits and the proportion unemployed). We also made sure that dropping variables did not entail major changes in coefficients of the retained variables, which would suggest the presence of omitted variable bias.

Having arrived at the main FD model, we then conducted model diagnostics and carried additional modelling of its variants, to address potential violation of model assumptions.

## Results

Table 2 presents results of the main FD model. The outcome variable was the number of food parcels distributed by the food banks in the Trussell Trust network per 1000 working age (WA) population in a financial year.

**Table 2:** Results of a FD regression model predicting food parcel uptake, 309 local authorities in England, 2011/12–2019/20

	Coef.	Robust Std. Err.	Significance (*p*-value)	95% Conf. Interval
Number of food bank centres in the Trussell Trust network per 1000 WA^a^ population	358.30	27.80	0.000	303.78,412.82
Percent of WA population who are unemployed	0.85	0.34	0.013	0.18,1.51
Real value of main income replacement benefit^b^	−1.37	0.26	0.000	−1.89,-0.85
Percent of WA population on out-of-work benefits	−2.97	0.68	0.000	−4.31,-1.63
Interaction of the two preceding variables	−0.62	0.65	0.346	−1.90,0.67
Percent of claimants of WA benefits who are on UC	0.36	0.04	0.000	0.27,0.44
Number of JSA and ESA sanctions per 1000 WA population	0.24	0.05	0.000	0.13,0.34
Number of households affected by ‘bedroom tax’ per 1000 WA population	0.46	0.14	0.001	0.19,0.73
Constant	0.61	0.36	0.092	−0.10,1.32

Observations = 2472

R-squared = .31

^a^WA: working age

^b^UC/JSA/ESA/IS standard or personal allowance for people aged 25 or above. Weekly value adjusted for inflation. Reference year: 2011

16 local authorities were dropped due to values missing on one of the independent variables

The first predictor in Table 2, the number of the food banks in the Trussell Trust network per 1000 working age population, controls for the size of the potential ‘supply’ of food parcels. The coefficient means that on average, an increase in the number of food bank centres by one in the local authority is associated with 358 additional food parcels per year, an 8% year-on-year increase in a typical local authority (relative to the 2019/20 level). As an increase in the number of food bank centres by one means, on average, an increase of 28% in the number of food bank centres,^1^ the effect of this additional ‘supply’ is much less than proportional.

The next predictor, unemployment rate, shows that a one percentage point higher unemployment would have led to 0.85 more food parcels per 1000 working age population, equivalent to an extra 107 in a typical local authority, a 2% increase in the number of food parcels distributed. This suggests that food bank need was not very strongly driven by unemployment in this period.

The following predictor is the standard allowance of the main income replacement benefit (currently UC, previously Jobseeker’s Allowance), adjusted for inflation. The coefficient is negative, meaning that an increase in the real value of this allowance is associated with a decrease in the uptake of food parcels: the more income benefit claimants have, the less they use food banks. A £1 per week increase in UC standard allowance (in 2011 pound value) was associated with an annual decrease of 2.6%, or 118 food parcels in a typical local authority.^2^ This provides evidence that the real terms reduction in the basic working age benefit allowances has been an important factor in increasing food bank need.

The next predictor refers to the proportion of working age population who are in receipt of out-of-work benefits, that is UC (out-of-work parts only), Jobseeker’s Allowance, Employment and Support Allowance, or Income Support. One percentage point more of the working age population on such benefits would have reduced food parcels by 2.7 per 1000 working age population, 291 per year in a typical local authority, or about 6.5% of the 2019/20 level. While the coefficient’s negative sign may seem somewhat unintuitive, one needs to consider that some people are excluded from the welfare safety net because of their immigration status, while some others struggle to apply for benefits or to sustain the benefit claim [1]. Having stable benefit income usually protects from destitution and food bank use.

The following predictor is an interaction of the preceding two. Although it is not statistically significant, it is included in the model for a substantive reason: the value of the main out-of-work benefit will have stronger impact in local authorities with a larger proportion of people on out-of-work benefits. The interaction has the expected negative sign.

Next, an increase of 10 percentage points in those receiving UC as proportion of all claimants of working age benefits, was associated with an annual increase of 3.6 per 1000 in the number of food parcels, 454 in a typical local authority, an increase of 8.4% on the 2019/20 level. This indicates that the rollout of UC has been a major driver of increased food bank need.

An increase of 100 in the number of benefit sanctions was associated with an annual increase of 24 in the number of parcels.

Lastly, an increase in the number of households subject to ‘bedroom tax’ of 100 was associated with an annual increase in the number of food parcels of 46.

Overall, these predictors explain approximately 31% of the variance of annual increase in food bank use. While this is a respectable figure by social research standards, clearly the model does not provide a complete picture of drivers of food bank use, although we tested a wide range of variables.

Model diagnostic procedures have shown that the model fit is satisfactory (see Additional file 4 for details).

## Discussion

In the situation where conducting a randomised control experiment into the role of welfare policies in driving food bank use is impossible, findings presented in the previous section constitute, in our view, robust quasi-experimental evidence that four aspects of the welfare system partly drove the uptake of food parcels in the UK between 2011/12–2019/20. These elements were: the reducing value of the standard allowance received by claimants of out-of-work benefits; benefit sanctions; the roll-out of UC; and ‘bedroom tax’ in social housing. The finding about benefit sanctions independently verifies the result of two earlier studies [40, 41], while the finding about the other three aspects is an original contribution of this study.^3^

The implications of this evidence are three-fold. On an evidential level, it strongly implies that the current welfare system in the UK is partly responsible for generating food bank use. Since 2010, the structure of this system has been deliberately changed to provide overall less support to working age claimants [52]. While this has been primarily justified by the need to reduce state deficit, a more ideological agenda focused on the need to reduce apparent welfare dependency and incentivise paid employment has also arguably been central to the aims of the reforms [34].

Secondly, on the level of social and political action directly relevant to the nutritional condition of the population this evidence means that in order to reduce food bank use, the UK would need to reform or amend welfare policies that have been evidenced to drive it. The evidence presented here can be exploited to prioritise reforms, by identifying effects of different potential reforms on the uptake of food parcels. We have carried out additional policy forecasting [53] - based on the main model and assuming the Covid-19 pandemic has not happened - which indicated that retaining the £20/week uplift to UC (introduced in April 2020 and ended in October 2021) would result in the largest reduction of the uptake, followed by suspending the ‘bedroom tax’, suspending benefit sanctions and finally halting the intake of new claimants onto UC. The intake of new claimants onto UC obviously cannot be halted, but the most destitution-generating aspects of UC can be reformed (the initial five-week wait in particular, but also debt recovery arrangements and problems with applying online; see [48, 49]).

It needs to be remembered, however, that the list of social policies creating demand for food parcels might not be limited to the four measures evidenced here. A large volume of qualitative and descriptive quantitative evidence exists pointing at the role of PIP assessments, Work Capability Assessments, low LHA rates, Benefit Cap, and the two-child limit (among other policies) in driving food bank use (and destitution in general; see [49]). Some of these factors have been explored in the modelling but dropped from our final model due to lack of statistical significance. Lack of statistical significance results either from insufficient volume of data (while the effect is genuine) or due to there not being any effect. The above-mentioned existing evidence suggests to us that in this case the lack of significance could be due to insufficient amount of data.

Thirdly, the evidence in this paper provides support for ‘Health in All Policies’ approach to policymaking. Health in All Policies is an approach to all public policies (whether health related or not) that systematically takes into account the health implications of decisions, seeks synergies, and avoids harmful health impacts in order to improve population health and health equity [54]. The rationale for this approach lies in recognition of the fact that ‘health, wellbeing and health inequalities [ …] are largely determined by living conditions and wider social, economic, environmental, cultural and political factors. These in turn are controlled by policies and actions outside the health sector’ [55]. Therefore, policymaking that is not explicitly health-focused but has potential negative health implications needs to be aware of these implications and take them into account in the cost-benefit analysis of the prospective policy. The fact that the Westminster Government’s own impact assessment of the ‘bedroom tax’ states that the policy would not have impact on health and well-being [56] is a case in point.

### Strengths and limitations

As for the strengths of the current analysis, we believe that the choice of methodological approach has been robust in current circumstances. While instrumental variables and regression discontinuity are quasi-experimental techniques that may be theoretically superior to the ones employed here, we have not found it practically possible to make use of these.

Another strength of the analysis is that the results are in close agreement with evidence from countries similar to the UK, such as the US, Canada, Finland and Germany. That evidence suggests that food banks may appear on some scale where recessions trigger a rise in unemployment, but it also shows that the number of food banks increased sharply when very weak economic position of low-income households was accompanied by weakening of the safety net [11, 57, 58]. In the US, the first food banks appeared in the 1960s but their number soared in early 1980s, due to a combination of economic recession and welfare cuts introduced by Reagan’s administration [11, 59] showed that The Personal Responsibility and Work Opportunity Reconciliation Act of 1996 (PRWORA) impacted on low-income immigrant households so much that it continued driving their demand for food aid even when the overall economy was growing in the late 1990s [60]. similarly demonstrated that for those affected by PRWORA, a decline in the reach of cash assistance combined with increased welfare conditionality resulted in increased demand for food aid – even before the economic crisis of 2008. In the case of Finland, the recession of the early 1990s on its own was enough to bring about ‘bread lines’, but as [58] argues it was the freezing of basic welfare benefits levels that led to food banks becoming common. In Germany, the number of food banks rose more steeply after the welfare reform of 2005 than after the global financial crisis of 2008 [61, 62].

What also increases our confidence in the validity of our findings is that they are in agreement with qualitative evidence collected by earlier UK studies [33, 35, 63], by the *State of Hunger* study itself [1], and – very importantly – with the recent Family Resources Survey (FRS) data. The 2019/20 edition of FRS shows that while severe food insecurity was experienced by 4% of all households, it was experienced by 26% of households claiming UC, 14% of households on any income-related benefit, and 17% of households in the social rented sector (where ‘bedroom tax’ applies) [5]. This clearly chimes with our analysis pointing at low out-of-work benefit levels, the roll-out of Universal Credit, and the ‘bedroom tax’.

Existing qualitative evidence not only provides a sense-check of our quantitative findings, but it also fleshes out those findings that are by themselves ‘bare’. In particular, the finding about the driving role of the UC roll-out tells us nothing regarding what it is about UC that specifically generates destitution. Qualitative evidence, in contrast, unequivocally points at the initial five-week wait for the first UC payment as the key aspect of UC design that has been driving food bank use [63, 64], as well as at the arrangements for advance and debt repayments.

A limitation of the study is that due to its design it could not fully investigate the driving role of factors identified by the *State of Hunger* study as ‘background’ drivers of food bank use: aspects of ill health, adverse life events (such as household breakdown), and lack of support. (These kinds of factors are ‘background’ in the sense of them contributing to the household being more financially vulnerable, while a reduction or loss of benefit income – or a long wait for it - is an ‘immediate’ driver of food bank use [1]. Such ‘background’ factors are best examined through use of micro survey data - preferably longitudinal in form – rather than data about aggregates of people, such as local authorities). Therefore, the analysis presented here covers only part of the wider picture of drivers. This may be reflected in the R-squared from the main model having a value of 0.31, which is respectable for an FD model, but clearly a large proportion of the variance of annual increase in food bank use is not explained by this model.

Lastly, it is a limitation of the study that it covered only food banks in the Trussell Trust network. Therefore, our findings correspond only to certain food banks (albeit a numerical majority) and may not be replicated in food banks operating independently of the Trussell Trust.

## Conclusions

Food bank use is a seriously concerning phenomenon from the public health perspective. The vast majority of food bank users are severely food insecure, which signifies inadequate diet. Inadequate diet has been linked to a range of negative physical and mental health outcomes. Hence, the increase in in people using food banks over the 2010s calls for an action to alleviate the level of severe food insecurity in the population, as manifested by food bank use. This however requires sound, evidence-based understanding of factors that have been driving food bank use in the UK in the last decade. This paper provided quasi-experimental evidence that four aspects of the welfare benefit system have been driving food bank use: falling value of welfare benefits, the structure of UC, the ‘bedroom tax’ in social housing, and benefit sanctions. These findings mean that one way to improve food security would be to reform the welfare benefit system through a combination of increasing the minimum value of benefits, reshaping UC (particularly shortening the initial waiting period), suspending or lessening the ‘bedroom tax’ and suspending or lessening benefit conditionality. Going beyond the immediate focus of this research, the findings also suggest that health inequalities in England could be reduced, and health outcomes improved, if ‘Health and Health Equity in All Policies’ approach was adopted by policymakers.

## Supplementary Information

**Additional file 1.** Variables considered for the modelling.

**Additional file 2.** Omitted variable bias.

**Additional file 3.** Dynamics.

**Additional file 4.** Model Diagnostics.

## Data Availability

The master dataset is not publicly available but can be recreated (see Additional file 2). All but one subsidiary dataset feeding into the master dataset are publicly available. Access to the data about the number of food parcels distributed by food banks belonging to the Trussell Trust network in each Local Authority in each financial year would need to be negotiated with the Trussell Trust. The Stata syntax is available from the corresponding author upon request.

## Abbreviations

ESA: Employment and Support Allowance
FD: First Differencing
FRS: Family Resources Survey
FE: Fixed Effects
IS: Income Support
JSA: Jobseeker’s Allowance
LHA: Local Housing Allowance
PIP: Personal Independence Payment
PRWORA: The Personal Responsibility and Work Opportunity Reconciliation Act
UC: Universal Credit
UK: United Kingdom
US: United States of America
WA: Working Age
WR: Welfare Reform

## Footnotes

1 In 2019/20, the mean number of food banks per local authority was 3.6.

2 Note that the figure of 2.6% cannot be calculated from Table 2 alone because it is the outcome of the main effect (real value of main income replacement benefit in a given year) plus its interaction with the average proportion of WA population on out-of-work benefits, across all local authorities, in a given year. An analogical comment applies to the figure of 2.7 in the following paragraph.

3 The finding about the driving role of UC has been subsequently independently verified by [51].
