# Supplementary material for: Understanding the post-2010 increase in food bank use in England: new quasi-experimental analysis of the role of welfare policy
Source: BMC Public Health. 2022 Jul 16;22:1363. doi: 10.1186/s12889-022-13738-0 (PMC9287534; doi:10.1186/s12889-022-13738-0)
Supplement: Supplementary file 1 — Additional file 1. Variables considered for the modelling. [file 12889_2022_13738_MOESM1_ESM.docx]

Additional file 1: Variables considered for the modelling

Variables marked with an asterix were divided by the size of the working age population.

Inflation data used to calculate the real-term value was downloaded from the ONS website.^[[1]](#footnote-1)^

The size of the working age population was sourced from NOMIS (dataset ‘Population estimates – local authority based by single year of age’; https://www.nomisweb.co.uk/query/construct/summary.asp?mode=construct&version=0&dataset=2002).

| **Variable** | **Source** | **Shortlisted** | **Retained in the main model** |
| --- | --- | --- | --- |
|  |  |  |  |
| ***Demographic controls and other controls:*** |  |  |  |
| - number of operational Trussell Trust food bank centres* | The Trussell Trust administrative system | Yes | Yes |
| - number of lone parent households* | Annual Population Survey – Households with dependent children and type (from NOMIS; https://www.nomisweb.co.uk/query/construct/summary.asp?mode=construct&version=0&dataset=137) |  |  |
| - number of people who are non-UK born* | Annual Population Survey (from NOMIS; https://www.nomisweb.co.uk/query/construct/summary.asp?mode=construct&version=0&dataset=17) |  |  |
| - number of working age people who have a disability that limits the amount or the kind of work that they can do* | Annual Population Survey (from NOMIS; https://www.nomisweb.co.uk/query/construct/summary.asp?mode=construct&version=0&dataset=17) | Yes |  |
|  |  |  |  |
| ***Economy-related variables:*** |  |  |  |
| - real gross weekly median pay (full-time workers) | Annual Survey of Hours and Earnings (from NOMIS; https://www.nomisweb.co.uk/query/construct/summary.asp?mode=construct&version=0&dataset=30) |  |  |
| - real gross weekly pay at 10^th^ percentile (full-time workers) | Annual Survey of Hours and Earnings (from NOMIS; https://www.nomisweb.co.uk/query/construct/summary.asp?mode=construct&version=0&dataset=30) |  |  |
| - percent of employees working on a part-time basis | Annual Population Survey (from NOMIS; https://www.nomisweb.co.uk/query/construct/summary.asp?mode=construct&version=0&dataset=17) |  |  |
| - jobs density^[[2]](#footnote-2)^ | Jobs Density (from NOMIS; https://www.nomisweb.co.uk/query/construct/summary.asp?mode=construct&version=0&dataset=57) |  |  |
| - number of work seekers (number of JSA claimants combined with the number of UC claimants in the ‘searching for work’ category)* | Stat-Xplore (<https://stat-xplore.dwp.gov.uk/>; datasets ‘Jobseekers Allowance’ and ‘People on Universal Credit’) |  |  |
| - real value of specific parts of local authority budgets (homelessness, Supporting People, mental health), £ per capita | CIPFA (https://www.cipfa.org/) |  |  |
| - real value of main out-of-work benefits (JSA/ESA/IS personal allowance; UC standard allowance). The reference year was 2011. | McInnes (2019) *Benefits Uprating 2019*, Briefing Paper Number CBP 8458, London: House of Commons Library. | Yes | Yes |
| - percent of working age population who are unemployed | Model-based estimates of unemployment (from NOMIS; https://www.nomisweb.co.uk/query/construct/summary.asp?mode=construct&version=0&dataset=127) | Yes | Yes |
| - percent of working age population on out-of-work benefits | Stat-Xplore (<https://stat-xplore.dwp.gov.uk/>; dataset ‘Benefit Combinations’) | Yes | Yes |
|  |  |  |  |
| ***Housing-related variables:*** |  |  |  |
| - real private rent at 25^th^ percentile (three versions: room, 1 bed, 2 bed), £ per month | Valuation Office Agency (https://www.gov.uk/government/collections/private-rental-market-statistics) |  |  |
| - number of non-passported HB claimants (a proxy for HB not covering full rent)* | Stat-Xplore (<https://stat-xplore.dwp.gov.uk/>; dataset ‘Housing Benefit’) |  |  |
| - number of PRS LHA claimants* | Stat-Xplore (<https://stat-xplore.dwp.gov.uk/>; dataset ‘Housing Benefit’) |  |  |
| - discrepancy between the value of LHA and real private rent at 25^th^ percentile, £ per month (three versions: room, 1 bed, 2 bed) | LHA rates from Valuation Office Agency (https://www.gov.uk/government/collections/local-reference-rents-levels-collection). Real private rent data from Valuation Office Agency (https://www.gov.uk/government/collections/private-rental-market-statistics). |  |  |
| - Council Tax collected by LA as proportion of all collectible CT (a proxy for Council Tax arrears) | MHCLG (https://www.gov.uk/government/collections/council-tax-statistics#collection-rates-for-council-tax-and-non-domestic-rates) |  |  |
| - number of SRS households on HB* | Stat-Xplore (<https://stat-xplore.dwp.gov.uk/>; dataset ‘Housing Benefit’) |  |  |
|  |  |  |  |
| ***Homelessness-related variables:*** |  |  |  |
| - number of households accepted as homeless* | MHCLG (https://www.gov.uk/government/statistical-data-sets/live-tables-on-homelessness) | Yes |  |
| - number of single persons accepted as homeless* | MHCLG (https://www.gov.uk/government/statistical-data-sets/live-tables-on-homelessness)  ‘No priority need’ was used as a proxy for single applicants. |  |  |
| - number of households in Temporary Accommodation* | MHCLG (https://www.gov.uk/government/statistical-data-sets/live-tables-on-homelessness) | Yes |  |
|  |  |  |  |
| ***Welfare-related variables:*** |  |  |  |
| - number of claimants of ESA/IB/SDA/UC 'no work requirement'/UC 'preparing for work'* | Stat-Xplore (<https://stat-xplore.dwp.gov.uk/>; dataset ‘Benefit Combinations’) |  |  |
| - number of cases of failed DLA to PIP reassessment* | Stat-Xplore (<https://stat-xplore.dwp.gov.uk/>; dataset ‘DLA to PIP Reassessments’) | Yes |  |
| - number of cases of unsuccessful fresh PIP assessment* | Stat-Xplore (<https://stat-xplore.dwp.gov.uk/>; dataset ‘PIP Clearances’) | Yes |  |
| - number of households subject to ‘bedroom tax’* | Stat-Xplore (<https://stat-xplore.dwp.gov.uk/>; dataset ‘Housing Benefit’) | Yes | Yes |
| - number of households subject to Benefit Cap* | Stat-Xplore (<https://stat-xplore.dwp.gov.uk/>; dataset ‘Benefit Cap’) |  |  |
| - number of JSA/ESA/IS sanctions* | Stat-Xplore (<https://stat-xplore.dwp.gov.uk/>; dataset ‘Sanction Decisions’) | Yes | Yes |
| - number of UC sanctions* | Stat-Xplore (<https://stat-xplore.dwp.gov.uk/>; dataset ‘Sanction Decisions’) |  |  |
| - percent of UC claimants among all working age benefit claimants | Stat-Xplore (<https://stat-xplore.dwp.gov.uk/>; dataset ‘Benefit Combinations’) | Yes | Yes |
| - number of ‘fit for work’ outcomes of WCA* | Stat-Xplore (<https://stat-xplore.dwp.gov.uk/>; dataset ‘ESA Work Capability Assessments’) |  |  |
| - number of LHA recipients subject to SAR* | Stat-Xplore (<https://stat-xplore.dwp.gov.uk/>; dataset ‘Housing Benefit’) | Yes |  |
| - number of SRS tenants with HB paid to claimant instead of the landlord* | Stat-Xplore (<https://stat-xplore.dwp.gov.uk/>; dataset ‘Housing Benefit’) |  |  |
| - number of households subject to HB non-dependent deductions (three versions: 1+, 2+, 3+ non-dependents)* | Stat-Xplore (<https://stat-xplore.dwp.gov.uk/>; dataset ‘Housing Benefit’) | Yes |  |

1. https://www.ons.gov.uk/economy/inflationandpriceindices/datasets/consumerpriceindices [↑](#footnote-ref-1)
2. The number of jobs in an area divided by the resident population aged 16-64 in that area. For example, a job density of 1.0 would mean that there is one job for every resident aged 16-64. [↑](#footnote-ref-2)
