# Supplementary material for: Understanding the post-2010 increase in food bank use in England: new quasi-experimental analysis of the role of welfare policy
Source: BMC Public Health. 2022 Jul 16;22:1363. doi: 10.1186/s12889-022-13738-0 (PMC9287534; doi:10.1186/s12889-022-13738-0)
Supplement: Supplementary file 2 — Additional file 2. Omitted variable bias. [file 12889_2022_13738_MOESM2_ESM.docx]

## Additional file 2: Omitted variable bias

Two additional variants of the main model were developed to address potential omitted variable bias. Firstly, data about the number of independent food banks (i.e. not in the Trussell Trust network) in each local authority was added to the model. Relevant longitudinal data does not exist, but the research team had access to cross-sectional data from 2019 [1]. These data were included in the model in the form of a weight, which - to some extent - fed into the model information about the relevant part of the error term. The fact that regression coefficients are slightly different in the weighted model, in comparison to the unweighted one, means that the weight and the error term are correlated, confirming the supposition about the outcome being influenced by the number of independent food banks. As can be seen in Table A2.1, the coefficient on ‘the number of food bank centres in the Trussell Trust network’ decreased, which is in agreement with an argument about independent food banks exerting downwards pressure on the uptake of food parcels among food banks in the Trussell Trust network. However, other coefficients from the weighted model are very close to those from the main model, increasing our confidence in the validity of the main model.

Table A2.1 Results of a weighted FD regression model predicting food parcel uptake, 309 local authorities in England, 2011/12-2019/20

|  | Coef. | Robust Std. Err. | Significance (p-value) | 95% Conf. Interval |
| --- | --- | --- | --- | --- |
| Number of food bank centres in the Trussell Trust network per 1,000 WA* population | 347.82 | 28.20 | 0.000 | 292.51,403.13 |
| Real value of main income replacement benefit** | -1.40 | 0.28 | 0.000 | -1.95,-0.85 |
| Percent of WA population on out-of-work benefits | -3.13 | 0.73 | 0.000 | -4.57,-1.69 |
| Interaction of the two preceding variables | -0.68 | 0.71 | 0.336 | -2.07,0.71 |
| Percent of WA population who are unemployed | 0.89 | 0.36 | 0.014 | 0.18,1.61 |
| Percent of claimants of WA benefits who are on UC | 0.38 | 0.04 | 0.000 | 0.29,0.47 |
| Number of JSA and ESA sanctions per 1,000 WA population | 0.26 | 0.06 | 0.000 | 0.15,0.38 |
| Number of households affected by ‘bedroom tax’ per 1,000 WA population | 0.53 | 0.15 | 0.001 | 0.23,0.83 |
| Constant | 0.69 | 0.39 | 0.077 | -0.07,1.45 |

Observations = 2,464

R-squared = 0.32

* WA: working age

** UC/JSA/ESA/IS standard or personal allowance for people aged 25 or above. Weekly value adjusted for inflation. Reference year: 2011.

The second attempt at neutralizing potential omitted variable bias took the form of adding to the model a spatial lag of the outcome variable (i.e., weighted averages of observations for the ‘neighbours’ of a given location, based on contiguity). This to some extent controlled for unobserved time-varying local characteristics, of which the presence of independent food banks is one case [2]. The effectiveness of this approach relies on an assumption that neighbouring panel members are similar in terms of unobserved characteristics. This assumption holds for the presence of independent food banks: the number of independent food banks among neighbours is associated with the number of independent food banks in a given local authority. As can be seen in Table A2.2, the coefficients in this model are again close to coefficients in the main model reported in Table 2.

One technical difficulty with spatial lag models is that the spatial lag of the outcome introduces endogeneity. To address this, it is necessary to use a second-order spatial lag (weighted averages of observations for the ‘neighbours of neighbours’ of a given location) as an instrument for the first-order spatial lag.

Table A2.2 Results of a FD regression model predicting food parcel uptake, 309 local authorities in England, 2011/12-2019/20

|  | Coef. | Robust Std. Err. | Significance (p-value) | 95% Conf. Interval |
| --- | --- | --- | --- | --- |
| Spatial lag of the outcome variable (contiguity matrix) | 0.06 | 0.17 | 0.723 | -0.27,0.39 |
| Number of food bank centres in the Trussell Trust network per 1,000 WA* population | 357.02 | 29.73 | 0.000 | 298.74,415.29 |
| Real value of main income replacement benefit** | -0.86 | 0.53 | 0.103 | -1.89,0.17 |
| Percent of WA population on out-of-work benefits | -1.27 | 3.63 | 0.727 | -8.38,5.85 |
| Interaction of the two preceding variables | -0.03 | 0.06 | 0.668 | -0.15,0.10 |
| Percent of WA population who are unemployed | 0.84 | 0.3 | 0.005 | 0.25,1.43 |
| Percent of claimants of WA benefits who are on UC | 0.36 | 0.04 | 0.000 | 0.28,0.44 |
| Number of JSA and ESA sanctions per 1,000 WA population | 0.26 | 0.05 | 0.000 | 0.16,0.35 |
| Number of households affected by ‘bedroom tax’ per 1,000 WA population | 0.35 | 0.16 | 0.03 | 0.03,0.67 |
| Constant | 0.55 | 0.63 | 0.389 | -0.69,1.79 |

Observations = 2,472

R-squared: within 0.54, between 0.07, overall 0.31. Rho: 0.47.

* WA: working age

** UC/JSA/ESA/IS standard or personal allowance for people aged 25 or above. Weekly value adjusted for inflation. Reference year: 2011.

**References**

[1] Independent Food Banks Map. *Independent Food Aid Network*, https://www.foodaidnetwork.org.uk/independent-food-banks-map (accessed 19 July 2019).

[2] Duranton G, Gobillon L, Overman HG. Assessing the Effects of Local Taxation using Microgeographic Data. *Econ J* 2011; 121: 1017–1046.
