# Supplementary material for: Understanding the post-2010 increase in food bank use in England: new quasi-experimental analysis of the role of welfare policy
Source: BMC Public Health. 2022 Jul 16;22:1363. doi: 10.1186/s12889-022-13738-0 (PMC9287534; doi:10.1186/s12889-022-13738-0)
Supplement: Supplementary file 3 — Additional file 3. Dynamics. [file 12889_2022_13738_MOESM3_ESM.docx]

## Additional file 3: Dynamics

While we verified the absence of potential nonstationarity in the dependent variable, ignored stationary dynamics can also bias our findings. Hence, we fit a dynamic model, i.e. one that includes a lag of the outcome variable (the number of food bank centres in the Trussell Trust network) as one of the predictors. Specifically, an Arellano-Bond approach has been employed [1]. In this approach, initially first differencing is done to remove unobserved heterogeneity and then second- and higher order lags of the dependent variable~~s~~ are used as instruments to deal with endogeneity and the so-called Nickell bias [2]. Indeed, significant evidence of stationary dynamics is detected. However, coefficients on key predictors of interest (welfare policies) are very close to those in the main model, increasing our confidence in the robustness of those findings from the basic model.

[scroll down to the next page]

Table A3.1 Results of a dynamic (Arellano-Bond) regression model predicting food parcel uptake, 309 local authorities in England, 2011/12-2019/20

|  | Coef. | Robust Std. Err. | Significance (p-value) | 95% Conf. Interval |
| --- | --- | --- | --- | --- |
| First lag of the number of Trussell Trust food parcels per 1,000 WA population | 0.41 | 0.04 | 0.000 | 0.33,0.49 |
| Number of the Trussell Trust food bank centres per 1,000 WA* population | 103.98 | 52.76 | 0.049 | 0.56,207.39 |
| Real value of main income replacement benefit** | -0.86 | 0.54 | 0.109 | -1.92,0.19 |
| Percent of WA population on out-of-work benefits | 4.78 | 5.58 | 0.391 | -6.16,15.73 |
| Interaction of the two preceding variables | -0.08 | 0.07 | 0.283 | -0.22,0.06 |
| Percent of WA population who are unemployed | -0.22 | 0.88 | 0.804 | -1.94,1.50 |
| Percent of claimants of WA benefits who are on UC | 0.29 | 0.04 | 0.000 | 0.21,0.37 |
| Number of JSA and ESA sanctions per 1,000 WA population | 0.22 | 0.07 | 0.002 | 0.08,0.35 |
| Number of households affected by ‘bedroom tax’ per 1,000 WA population | 0.56 | 0.11 | 0.000 | 0.35,0.77 |

Observations = 2,163

Wald chi2: 1165.78, Prob > chi2: 0.000

* WA: working age

** UC/JSA/ESA/IS standard or personal allowance for people aged 25 or above. Weekly value adjusted for inflation. Reference year: 2011.

**References**

[1] Arellano M, Bond S. Some Tests of Specification for Panel Data: Monte Carlo Evidence and an Application to Employment Equations. *Rev Econ Stud* 1991; 58: 277–297.

[2] Leszczensky L, Wolbring T. How to Deal With Reverse Causality Using Panel Data? Recommendations for Researchers Based on a Simulation Study. *Sociol Methods Res* 2019; 004912411988247.
