# Supplementary material for: Understanding the post-2010 increase in food bank use in England: new quasi-experimental analysis of the role of welfare policy
Source: BMC Public Health. 2022 Jul 16;22:1363. doi: 10.1186/s12889-022-13738-0 (PMC9287534; doi:10.1186/s12889-022-13738-0)
Supplement: Supplementary file 4 — Additional file 4. Model Diagnostics. [file 12889_2022_13738_MOESM4_ESM.docx]

## Additional file 4: Model Diagnostics

A plot of residuals versus fitted values was inspected. The presence of a pattern would suggest that one or more assumptions have been violated. As can be seen in Figure 1, the plot appears pattern-less, particularly in the central area where there is a substantial number of observations. The fact that the lowess line slightly diverges from zero at the left and right extremes is not concerning, as the number of observations is small in those areas.

Figure 1 Plot of residuals versus fitted values

Wooldridge’s test of violations of strict exogeneity was also conducted [46]. The test suggested no violation in the case of six of the seven predictors. The predictor where the test suggested a potential violation was ‘the number of food bank centres in the Trussell Trust network’. This could be for two reasons, in our view:

(a) due to the existence of a time-varying unobserved confounder that is correlated with the number of food bank centres in the Trussell Trust network, resulting in omitted variable bias. The main suspect is the number of independent (non-Trussell Trust) food banks in the area, as the presence of such food banks is likely to imply downward pressure on the uptake of food parcels at food banks belonging to the Trussell Trust network. However, other factors – whether related to welfare policies or not, but not included in the model - could also create this effect.

(b) due to there being feedback from the number of food parcels distributed at one time point to the number of food banks in the next time point. It is possible that people involved in running a food bank, having observed substantial uptake of food parcels at their food bank, become encouraged to increase the number of food bank centres in the area. This would mean the presence of ‘reverse causality’.

A potential violation of the strict exogeneity assumption on that particular predictor does not imply that the rest of the model is untrustworthy; rather, it is the coefficient on that particular variable that may be misleading. Two additional variants of the main model were developed to address potential omitted variable bias. Both of these models had similar coefficients to the model reported in Table 2 (see Additional file 2 for more details).

While we verified the absence of temporal nonstationarity in the dependent variable, ignored stationary dynamics can also bias our findings. Hence, we fit a dynamic model, i.e. one that included a lag of the outcome variable as one of the predictors. While significant evidence of stationary dynamics was detected, coefficients on key predictors of interest (welfare policies) were very close to those in the main model (Table 2), increasing our confidence in the robustness of those findings from that main model (see Additional file 3 for more details).
